# A Quantitative Study of the Mechanisms behind Thymic Atrophy in Gαi2-Deficient Mice during Colitis Development

**DOI:** 10.1371/journal.pone.0036726

**Published:** 2012-05-10

**Authors:** Kristina Elgbratt, Andreas Jansson, Elisabeth Hultgren-Hörnquist

**Affiliations:** 1 School of Health and Medical Sciences, Örebro University, Örebro, Sweden; 2 Systems Biology Research Centre, University of Skövde, Skövde, Sweden; Charité, Campus Benjamin Franklin, Germany

## Abstract

Mice deficient for the G protein subunit Gαi2 spontaneously develop colitis, a chronic inflammatory disease associated with dysregulated T cell responses. We and others have previously demonstrated a thymic involution in these mice and an aberrant thymocyte dynamics. The Gαi2^−/−^ mice have a dramatically reduced fraction of double positive thymocytes and an increased fraction of single positive (SP) thymocytes. In this study, we quantify a number of critical parameters in order to narrow down the underlying mechanisms that cause the dynamical changes of the thymocyte development in the Gαi2^−/−^ mice. Our data suggest that the increased fraction of SP thymocytes results only from a decreased number of DP thymocytes, since the number of SP thymocytes in the Gαi2^−/−^ mice is comparable to the control littermates. By measuring the frequency of T cell receptor excision circles (TRECs) in the thymocytes, we demonstrate that the number of cell divisions the Gαi2^−/−^ SP thymocytes undergo is comparable to SP thymocytes from control littermates. In addition, our data show that the mature SP CD4^+^ and CD8^+^ thymocytes divide to the same extent before they egress from the thymus. By estimating the number of peripheral TREC^+^ T lymphocytes and their death rate, we could calculate the daily egression of thymocytes. Gαi2^−/−^ mice with no/mild and moderate colitis were found to have a slower export rate in comparison to the control littermates. The quantitative measurements in this study suggest a number of dynamical changes in the thymocyte development during the progression of colitis.

## Introduction

Inflammatory bowel disease (IBD) is a chronic relapsing inflammatory disorder of the gastrointestinal tract, comprising ulcerative colitis (UC) and Crohn's disease (CD). Defective regulation of T cell responses to the gut flora contributes to the aetiology of IBD and several mouse models for IBD are based on alterations in T lymphocyte subsets [1]–[2]. As T lymphocytes are shown to be one of the key cells involved in the aberrant immune responses in IBD it is of great interest to study the generation of new T lymphocytes in this setting.

In early age, thymus is the main organ for development of new naïve, immunocompetent T lymphocytes that will be exported to the periphery, seeding the peripheral lymphoid organs. Thymic atrophy begins from the time of sexual maturity, in both humans and mice [3], and the export of T lymphocytes from the thymus is then decreasing with age. Even though the total egression of thymocytes decreases with time, an export rate of 1–2% of the total thymocytes pool remains constant throughout life [3]–[4]. Acute thymic atrophy is a phenomenon induced by stress, pregnancy [5], infections or autoimmune disease [6] where the thymus in many case is later replenished and recovered [7], while thymic atrophy caused by age is irreversible.

The Gαi2^−/−^ mouse is a well-established model for colitis [8]–[10] with T helper type 1 (Th1) driven inflammation with increased levels of interferon-γ (IFN- γ), interleukin-1α (IL-1α), IL-6 and tumour necrosis factor- α (TNF- α) in inflamed tissue [10]. We and others have previously demonstrated a dramatic thymic involution in Gαi2^−/−^ mice with aberrant thymocyte development compared to control littermates [11]–[12]. The fraction of double positive (DP) thymocytes was severely reduced, resulting in an increased fraction of single positive (SP) thymocytes [11]–[12]. We have also found a similar aberrant thymocyte development in dextran sulphate sodium (DSS)-induced colitic mice [7]. In addition, IL-2 deficient mice challenged in vivo with TNP-KLH exhibit a thymocyte maturation defect [2]. Chemotaxis studies in Gαi2^−/−^ mice demonstrated an impaired capacity of thymocytes as well as colonic lamina propria lymphocytes to respond to CXCL12 compared to wild type mice [11]. Taken together, these observations suggest that the aberrant thymocyte development in mouse models of colitis might be a key event contributing to the development of colitis. A detailed study of the thymocyte development by Zhang et al. proposed an accelerated transition from DP to SP in Gαi2^−/−^ mice [12]. Since DP thymocytes proliferate vigorously before they develop into SP thymocytes one would expect that the reduced number of DP thymocytes would severely reduce the egression (export rate) of thymocytes from the thymus. In this study, we analysed the relative cell divisions taking place from the DP stage to the late SP stage as well as the dynamics in the periphery, which enabled us to estimate the thymic export rate in Gαi2^−/−^ mice. The thymic T lymphocyte production was estimated taking advantage of an intrinsic feature of the T cell receptor (TCR) rearrangement process that results in the generation of unique TCR excision circles (TRECs), a traceable molecular marker in newly produced T cells [13]–[14]. TRECs are stable and not duplicated during mitosis, and are thus diluted with each cell division [15]. The levels of TRECs in the periphery and the thymus can therefore be used to calculate the thymic export rate [16]. The method has been extensively used to study T cell reconstitution in highly active antiretroviral therapy (HAART) treated HIV-patients [17], thymic export function in relapsing remitting multiple sclerosis (RRMS) [18] and rheumatoid arthritis (RA) [19]–[20]. We have previously used estimation of TRECs levels to study the amount of recent thymic emigrants (RTEs) in the mucosa of IBD patients and found increased levels of RTEs in inflamed colons of UC patients compared to un-inflamed controls and CD patients [21]. Besides studies on thymus function in humans, TRECs have also been used in studies on aging mice [22], as well as studies on peripheral effects of IL-7 on RTEs in mice [23] and in experimental graft-vs-host disease (GVHD) [24]. Although these mouse models, including the Gαi2^−/−^ mice, involve increased numbers of activated T lymphocytes compared to the control littermates, only a small fraction of specific T lymphocytes are activated, which proliferate and are distributed to the site of inflammation. Hence, by studying only TREC^+^ T lymphocytes, the proliferating T lymphocyte clones is excluded from the calculation of the thymic egression. Hence, this method is therefore a reliable method to use for mouse models, such as the Gαi2^−/−^ mice, to analyze the thymic function and how this affects thymic egression during colitis development. The aim of this study was to quantify a number of critical parameters that might be affected during the onset of colitis in order to narrow down the possible mechanisms that may cause the aberrant thymocyte development seen in mouse models of colitis.

## Materials and Methods

### Ethics statement

All animals procedures were carried out under local and national ethical guidelines (Swedish Board of Agriculture) and were approved by the regional ethical committee, Gothenburg Administrative Court of Appeal, with the ethical approval ID 281-2010.

### Mice

Gαi2-deficient (Gαi2^−/−^) and healthy Gαi2^+/−^ control littermates on a pure 129SvEv background were used. The animals were kept at the Laboratory of Experimental Biomedicine, Göteborg University. Gαi2^−/−^ and Gαi2^+/−^ mice were bred using heterozygote males and females and the offspring were genotyped by polymerase chain reaction using tail genomic DNA. Gαi2^−/−^ mice develop a lethal colitis between four and seven weeks of age while Gαi2^+/−^ mice remain healthy. The latter is from now on referred as wild type (wt) or control mice. Gαi2^−/−^ and wt mice were sacrificed between four and nine weeks of age and Gαi2^−/−^ mice were scored macroscopically from 0 to 8p based on the following criteria: a) General (clinical) condition (0–4p); Passive/alert, presence/absence of “humpback” posture, ruff fur, soft faeces or diarrhoea, b) Visible signs of inflammation in colon (0–4p); colour (transparent or white) and thickness. They were then grouped into no/mild colitis; (0–2p), moderate colitis; (3–4p) or severe colitis; (5–8p). All mice were anaesthetized with isoflouran and then sacrificed by cervical translocation. As the kinetics of colitis development is similar in males and females, both sexes were used. All mice were maintained in micro-isolator racks with free access to water and rodent pellets in accordance with local and national ethical regulations and were health-screened in accordance with recommendations from the Federation of European Laboratory Animal Science Associations (FELASA), where they were confirmed to be specific pathogen free.

### Cell preparation

Mice were anaesthetized with isoflouran and blood was collected by retro-orbital puncture in tubes containing EDTA, before sacrifice. The blood volume was recorded and the blood was diluted 1∶4 in phosphate-buffered saline (PBS), and then stored on ice for maximum 1 h. Peripheral blood collected from Gαi2^+/−^ mice was pooled (3 mice/pool), as well as from two no colitis Gαi2^−/−^ mice while the blood from the rest of the Gαi2^−/−^ mice was analyzed individually. Peripheral blood mononuclear cells (PBMCs) were isolated by Ficoll/Hypaque (Amersham Biosciences AB, Uppsala, Sweden) density gradient centrifugation. The thymic lobes were dissected and pressed through a 100 µm nylon net. Thymocytes were then diluted in PBS and stored on ice for a maximum of 1 h. To enrich for the mature thymocyte population within the total thymocyte pool in wt mice, the cells were stained with anti-CD62L-conjugated magnetic beads (130-049-701, Miltenyi Biotec GmbH, Bergisch Gladbach, Germany) and separated twice on the positive selection program on an autoMACS Pro Separator (Miltenyi Biotec GmbH). PBMCs and thymocytes were washed twice in PBS and diluted in 6 ml RPMI-1640 medium containing 10% heat inactivated fetal calf serum (FCS) and then stored on ice for a maximum of 24 h.

### Flow cytometric analysis and cell sorting

Mature thymocytes were purified by flow cytometry: 2×10^6^ thymocytes per well in 5 wells were stained with the following antibodies: rat-anti-mouse-CD4-Phycoerythin-TexasRed (ECD) (GK1.5) (Abcam, Cambridge, UK), rat-anti-mouse-CD8-Phycoerythin-Cyanine-5 (PE-Cy5) (53-6.7) (Southern Biotech, Birmingham AL, US), hamster-anti-mouse-CD69-Phycoerythin (PE) (H1.2F3) (BD Biosciences Pharmingen, San Diego CA, US), and rat-anti-mouse-CD62L-Fluorescein isothiocyanate (FITC) (MEL-14) (BD Biosciences Pharmingen,), diluted in PBS 1% FCS up to a total volume of 30 µl and then incubated at 4°C for 20 min. For flow cytometric sorting of T lymphocytes in peripheral blood, 1–2×10^6^ PBMCs in a volume of 30 µl were stained with hamster-anti-mouse-CD3-PE (145-C211) (BD Biosciences Pharmingen). For analysis of naïve T lymphocytes, about 1×10^5^ PBMCs were stained with hamster-anti-mouse-CD3-PE (145-C211) and rat-anti-mouse-CD62L-FITC (MEL-14) (both from BD Biosciences Pharmingen). Analysis of the frequency of apoptotic naïve CD4^+^ T cells in peripheral blood was performed as follows; 1×10^5^ PBMCs were diluted in 100 µl Binding buffer (BD Biosciences Pharmingen) and then stained with rat-anti-mouse-CD4-ECD (Abcam), rat-anti-mouse-CD8-PE-Cy5 (Southern Biotech), AnnexinV-FITC and Propidium Iodide (both from BD Biosciences Pharmingen), incubated for 15 min at r.t. and then analysed within one hour. All flow cytometric analyses and cell sorting was performed on an EPICS® ALTRA™, (Beckman Coulter, Fullerton, CA, US). Mature thymocytes were gated and sorted as CD4^+^CD62L^+^(CD8^−^CD69^−/low^) and CD8^+^CD62L^+^(CD4^−^CD69^−/low^) cells, respectively and peripheral blood mononuclear cells were gated and sorted based on the expression of CD3. The purity of mature thymocytes was 90–98% and CD3^+^ PBMCs were 80–95% pure as analysed by the Kaluza version 1.1 software (Beckman Coulter).

### DNA extraction and real time-PCR for analysis of TRECs content

1–5×10^5^ sorted CD4^+^CD62L^+^ and CD8^+^CD62L^+^ thymocytes and CD3^+^ peripheral blood T lymphocytes were centrifuged, the supernatants were discarded. The pellets were diluted in 100 µl PBS, 100 µl Lysis buffer AL and 10 µl Proteinase K, and incubated at 56°C for 10 min according to the manufacturer's instructions (Qiagen, Hilden, Germany). The cell lysates were then stored at r.t. 1–2 weeks until DNA extraction. DNA was isolated and purified using the QIAmp DNA blood micro kit, according to the manufacturer's instructions (Qiagen). Prior to the real time-polymerase chain reactions (real time-PCR), DNA concentrations in all samples were determined using a NanoDrop® Spectrophotometer ND-1000 (NanoDrop Technologies, Wilmington, DE, US) at 260 and 280 nm wave lengths. The percentage of signal joint (sj) TRECs^+^ cells was identified by quantitative real time-PCR using a 7500 Real time-PCR System (Applied Biosystems, Warrington, UK). The DNA content from male and female laboratory mice cell nuclei have an estimated weight of 5.93 and 6.14 pg/nucleus, respectively (mean value calculated from 5 different strains; Balb/c, C57BL/6, CD1, C3H/heJ, and DBA and 10 different F1 hybrids crossed from these strains) [25]. Separate standard curves were set up, based on the DNA weight for male and female mice separately, starting with 59.3 ng and 61.4 ng, respectively, representing DNA from 1×10^4^ cells and were then diluted 10 times in each step for an additional 3 steps. To identify the number of cells in each sample in the standard curve, primer/probe was targeted for a unique DNA sequence within the glucagon gene, previously used as reference sequence [26]–[27]. The standard curve was run in parallel to the sample on the same plate and was used to calculate the percentage of sjTRECs in each sample based on the total amount of cells. Each 20 µl PCR reaction contained 10 µl FastMasterMix (Applied Biosystems), 2 µl of each primer (1.1 µM), 2 µl probe (0.25 µM) and 4 µl of sample DNA (1–20 ng/µl). The PCR was performed as follows: Initial step at 50°C for 2 min followed by 10 min initial denaturation at 95°C and subsequently 40 cycles of denaturation at 95°C for 15 sec followed by annealing/extension at 60°C for 1 min.

The following sequences of primers and probes were used: Glucagon sequence; forward primer 5′-CACAACATCTCGTGCCAGTCA-3′, reverse primer 5′-ATCTGCATGC AAAGCAATATAGCT-3′ and TaqMan MGB probe FAM-5′-GGGATGTACAATTTCAA-3′-TAMRA. SjTRECs sequence: forward primer 5′-CAGGGCAGGTTTTTGTAAAGGT-3′, reverse primer 5′-CCTGAGCATGGCAAGCAGTA-3′ and TaqMan probe FAM-5′-TGCTGTGTGCCCTACCCTGCCC-3′-TAMRA. All primers and probes were purchased from Invitrogen Life Technologies, Paisley, UK and Applied Biosystems, respectively.

### Calculations of the thymic export rate

By using the proposed kinetic model of peripheral TREC^+^ T cells by Hazenberg et al. [17]:
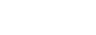
one can calculate the daily thymic export rate. The model is described as a differential equation, where dT/dt is the rate of change of TREC^+^ T cells in the periphery, *α* is the proportion of TREC^+^ cells exiting the thymus, *σ* is the export rate and *d* is the loss rate of TREC^+^ T cells. By assuming that the peripheral TREC^+^ T cells are at steady state (dT/dt = 0), the equation can be solved for the thymic export rate:
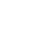
In this study we determined the fraction of TREC^+^ thymocytes within the most mature thymocyte populations (CD4^+^CD62L^+^ and CD8^+^CD62L^+^). This fraction corresponds to the parameter *α* in the model [16]. We also estimated the number of TREC^+^ T cells in the periphery, corresponding to the parameter 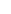, by counting the total number of TREC^+^ T cells in the blood and multiplying this number with 50, since 1/50 of all peripheral lymphocytes are located in the blood [16], [28]–[29]. The loss rate (*d*) of TREC^+^ T cells was taken from other studies, where a death rate of the majority of naïve T cells has been found to be 0,05/day in mice [28], [30].

## Results

### Unchanged amounts of single positive thymocytes in Gαi2^−/−^ mice during early colitis despite a dramatic reduction of double positive thymocytes

**Figure 1:**
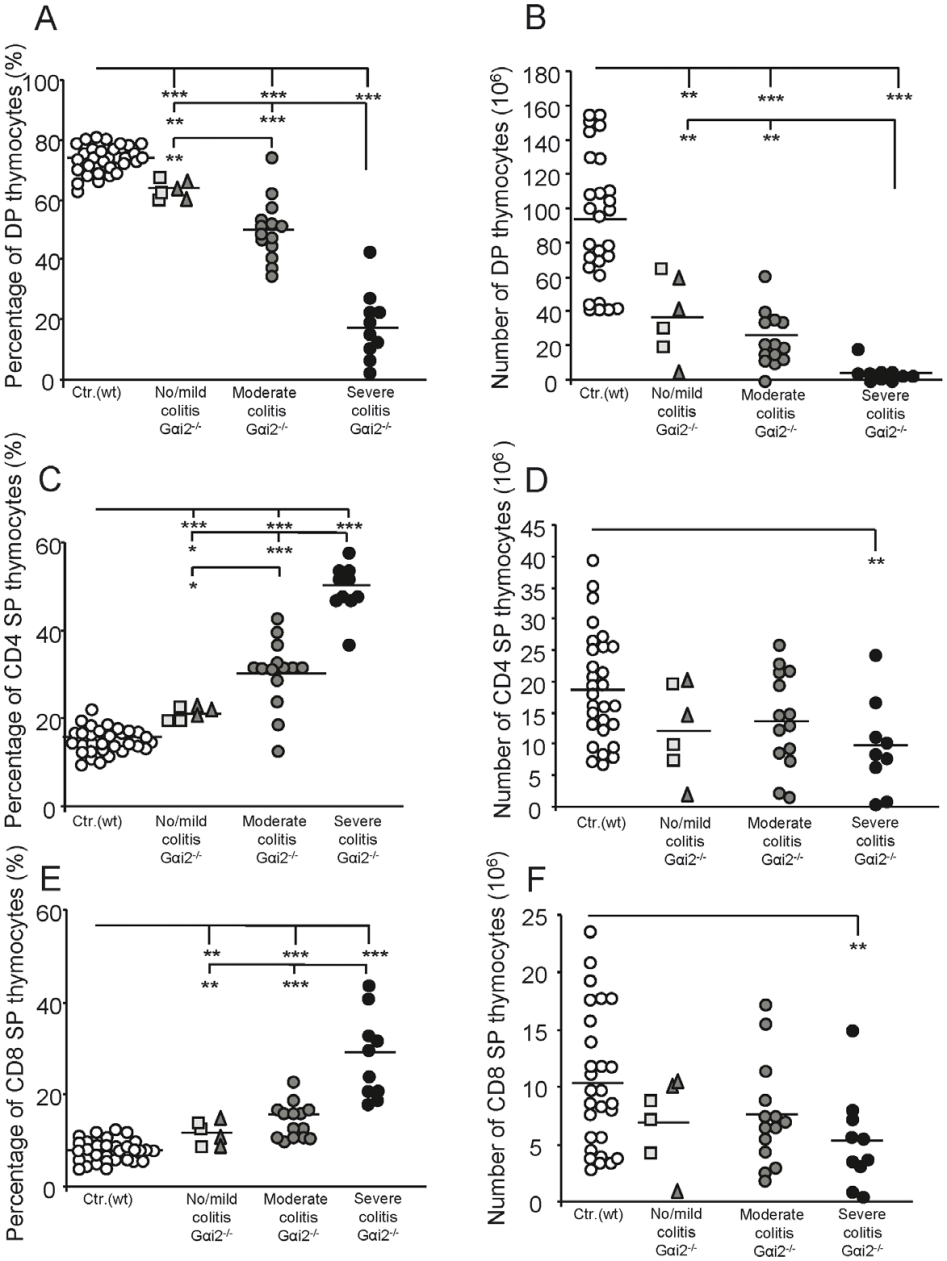
Decreased numbers and frequencies of DP thymocytes but equal numbers of SP thymocytes in Gαi2^−/−^ mice compared to control mice. Flow cytometric analyses of the frequencies and total numbers of CD4^+^8^+^ (DP) (A–B), CD4^+^8^−^ (CD4 SP) (C–D) and CD4^−^8^+^ (CD8 SP) (E–F) thymocytes. Results from wt mice (n = 28–31) and Gαi2^−/−^ mice (n = 6–14) are shown as frequencies or numbers of thymocytes from individual mice with the mean within each group presented as a horizontal line. The control group consisted of 4–9 weeks old wt mice whereas the Gαi2^−/−^ mice were 4–8 weeks old, grouped based on their colitis score; no and mild colitis (rectangles and triangles, respectively), moderate (dark gray circles) and severe colitis (black circles). Statistical analysis was performed using the Mann-Whitney non-parametric test and values of p≤0.05 were considered significant, *p≤0.05; **p≤0.01 and ***p≤0.001 between Gαi2^−/−^ mice and control mice or between Gαi2^−/−^ mice with different colitis scores.

Gαi2-deficient (Gαi2^−/−^) mice develop a severe thymic atrophy before any clinical symptoms of colitis are evident. To follow the progress of thymic atrophy, we first analysed the three main thymocyte populations; DP (CD4^+^CD8^+^), CD4 SP and CD8 SP thymocytes in wt mice based on their age (4–9 weeks). No significant changes in the numbers or fractions of DP or SP thymocytes were observed within the control (Gαi2^+/−^) littermate group based on their age: Age 4–5 w: 95.6±55.2×10^6^ DP, 18.3±11.6×10^6^ CD4 SP and 10.2±7.2×10^6^ CD8 SP; age 6–7 w: 107.8±10.5×10^6^ DP, 24.7±6.1×10^6^ CD4 SP and 13.7±5.1×10^6^ CD8 SP; 8–9 w: 72.4±6.1×10^6^ DP, 15.5±2.5×10^6^ CD4 SP and 7.5±2.9×10^6^ CD8 SP. The data were therefore collected into a single control group. We next analyzed the size of the major thymocyte population of the Gαi2^−/−^ mice compared to the control group. The Gαi2^−/−^ mice were first grouped according to their macroscopic colitis scores and the fraction and number of the different thymocyte populations (DP, CD4 SP and CD8 SP) was analyzed (Fig. 1). The mean frequency of DP thymocytes in Gαi2^−/−^ mice with no/mild colitis was significantly lower than in wt mice (Fig. 1A). As the colitis progressed, the frequency of DP thymocytes continued to decrease. The accelerated reduction of the DP thymocyte fraction in the Gαi2^−/−^ mice was also reflected in the total number of DP thymocytes. Gαi2^−/−^ mice with no/mild colitis had a mean value of ∼37×10^6^ DP thymocytes, which was significantly reduced compared to the ∼90×10^6^ DP thymocytes observed in the wt mice, and this reduction was even more pronounced in the more advanced stages of colitis (Fig. 1B). The frequency of CD4 and CD8 SP thymocytes increased gradually and significantly with the progression of colitis in Gαi2^−/−^ mice, compared to ∼16% of CD4 SP and ∼8% of CD8 SP thymocytes in the control mice (Fig. 1C & 1E). However, the total numbers of CD4 or CD8 SP thymocytes in Gαi2^−/−^ mice with no/mild and moderate colitis were comparable to the numbers in wt littermates (Fig. 1D & F). Hence, although the number of DP thymocytes decreased with progression of colitis, the numbers of SP thymocytes were comparable to the control littermates. However, mice with severe colitis showed significantly reduced numbers of both CD4 and CD8 SP thymocytes compared to the littermate controls. Next, we repeated this analysis with the Gαi2^−/−^ mice grouped according to age (4–8 weeks) instead of macroscopic scoring. A correlation analysis showed a stronger correlation between macroscopic colitis scores and the fraction (or number) of DP thymocytes of the total thymocytes compared to the correlation between age groups of Gαi2^−/−^ mice and the fraction (or number) of DP of the thymocytes (Table 1).

**Table 1:** Spearman's correlation analysis between age or colitis score and fraction or number of the different thymocyte subpopulations.

	Age^a^		Colitis score^b^	
Variable	*r*_s_	*p*	*r*_s_	*p*
% DP	−0.63	1.9•10^−4^	−0.85	2.7•10^−9^
% CD4	0.68	3.2•10^−5^	0.82	4.0•10^−8^
% CD8	0.58	8.7•10^−4^	0.77	4.8•10^−7^
# DP	−0.54	1.9•10^−3^	−0.72	5.9•10^−6^
# CD4	−0.17	0.37	−0.11	0.57
# CD8	−0.23	0.21	−0.23	0.31

a The different age groups were: 4 (n = 9), 5 (n = 7), 6 (n = 5), 7 (n = 6) and 8 weeks (n = 3).

b The different colitis score were: 0p (n = 3), 0.5–2p (n = 3), 2.5–4p (n = 14), 4.5–6p (n = 6) and 6.5–8p (n = 4).

### Comparable frequencies of TRECs^+^ mature thymocytes in wt and Gαi2^−/−^ mice indicate equivalent numbers of divisions during transition from DP to mature SP thymocytes

**Figure 2:**
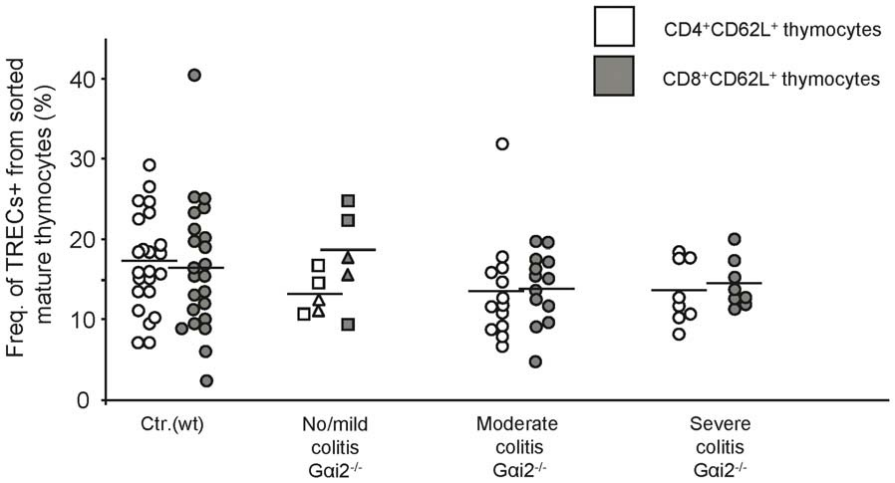
The frequencies of TRECs in mature thymocytes in Gαi2^−/−^ mice are independent of the colitis score. Mature CD4^+^CD8^−^CD62L^+^CD69^−^ and CD8^+^CD4^−^CD62L^+^CD69^−^ thymocytes from wt mice and Gαi2^−/−^ mice were sorted by flow cytometry. DNA from the sorted thymocytes were analysed for the amount of TRECs specific DNA by real time-PCR. The frequencies of TRECs^+^ cells in CD4^+^CD62L^+^ (white symbols) and CD8^+^CD62L^+^ (gray symbols) thymocytes were analysed in 4–9 weeks old wt mice (n = 28) and 4–8 weeks old Gαi2^−/−^ mice (n = 5–13), grouped based on colitis scores (rectangles = no colitis, triangles = mild colitis). The mean frequencies within each group are represented as a horizontal line. Statistical analysis was performed using the Mann-Whitney non-parametric test and values of p≤0.05 were considered significant.

As a part of the T lymphocyte maturation process in thymus, early DP thymocytes undergo an extensive rearrangement of the T cell receptor (TCR) genes, leaving circles of untranscribable DNA in the nucleus, one of which is termed signal joint T cell receptor excision circle (sjTREC). The sjTRECs are formed during the α-chain rearrangement at the developmental stage of DP thymocytes and remain stable within the cells. They are then diluted as the thymocytes undergo proliferation and develop into mature SP thymocytes. Knowing that Gαi2^−/−^ mice have an abnormal thymocyte composition compared to wt mice, we analysed whether the frequency of sjTREC^+^ mature thymocytes differed in Gαi2^−/−^ mice during development of colitis compared to wt littermates. Mature thymocytes were sorted based on the following phenotype: CD4^+^CD8^−^CD62L^+^CD69^−/low^ as well as CD8^+^CD4^−^CD62L^+^CD69^−/low^ (from now on referred to as CD4^+^CD62L^+^ and CD8^+^CD62L^+^ thymocytes, respectively) and analysed for the content of sjTRECs by quantitative real time-PCR. We first compared the frequencies of CD4^+^CD62L^+^ and CD8^+^CD62L^+^ thymocytes containing sjTRECs in 4 to 9 weeks old healthy wt mice; no significant differences in the frequencies of sjTRECs were found between the different age groups (age 4–5 w: 13.3±5.3% CD4^+^CD62L^+^ and 10.1±2.3% CD8^+^CD62L^+^; age 6–7 w: 18.9±4.7% CD4^+^CD62L^+^ and 15.4±3.8% CD8^+^CD62L^+^ and age 8–9 w: 18.3±6.7% CD4^+^CD62L^+^ and 20.6±9.8% CD8^+^CD62L^+^). We therefore pooled the data from the wt mice into a single control group. The wt mature CD4 SP and CD8 SP populations had comparable fractions of sjTREC^+^ thymocytes, indicating that in the wt mice the thymocytes undergo the same number of divisions from the α-chain rearrangement stage until they are mature SP thymocytes (Fig. 2). The sjTREC^+^ fractions of mature Gαi2^−/−^ thymocytes were similar in both the CD4^+^CD62L^+^ and CD8^+^CD62L^+^ populations, and did not significantly change during colitis development (Fig. 2). In addition, the fractions of sjTREC^+^ mature thymocytes were not significantly different between the control littermates and Gαi2^−/−^ mice. These results strongly indicate that the numbers of divisions the thymocytes undergo during the transition from DP to mature SP thymocytes are not different between Gαi2^−/−^ and Gαi2^+/−^ mice.

### Reduced numbers of TREC^+^ T lymphocytes in peripheral blood in mice with no/mild and moderate colitis

**Figure 3:**
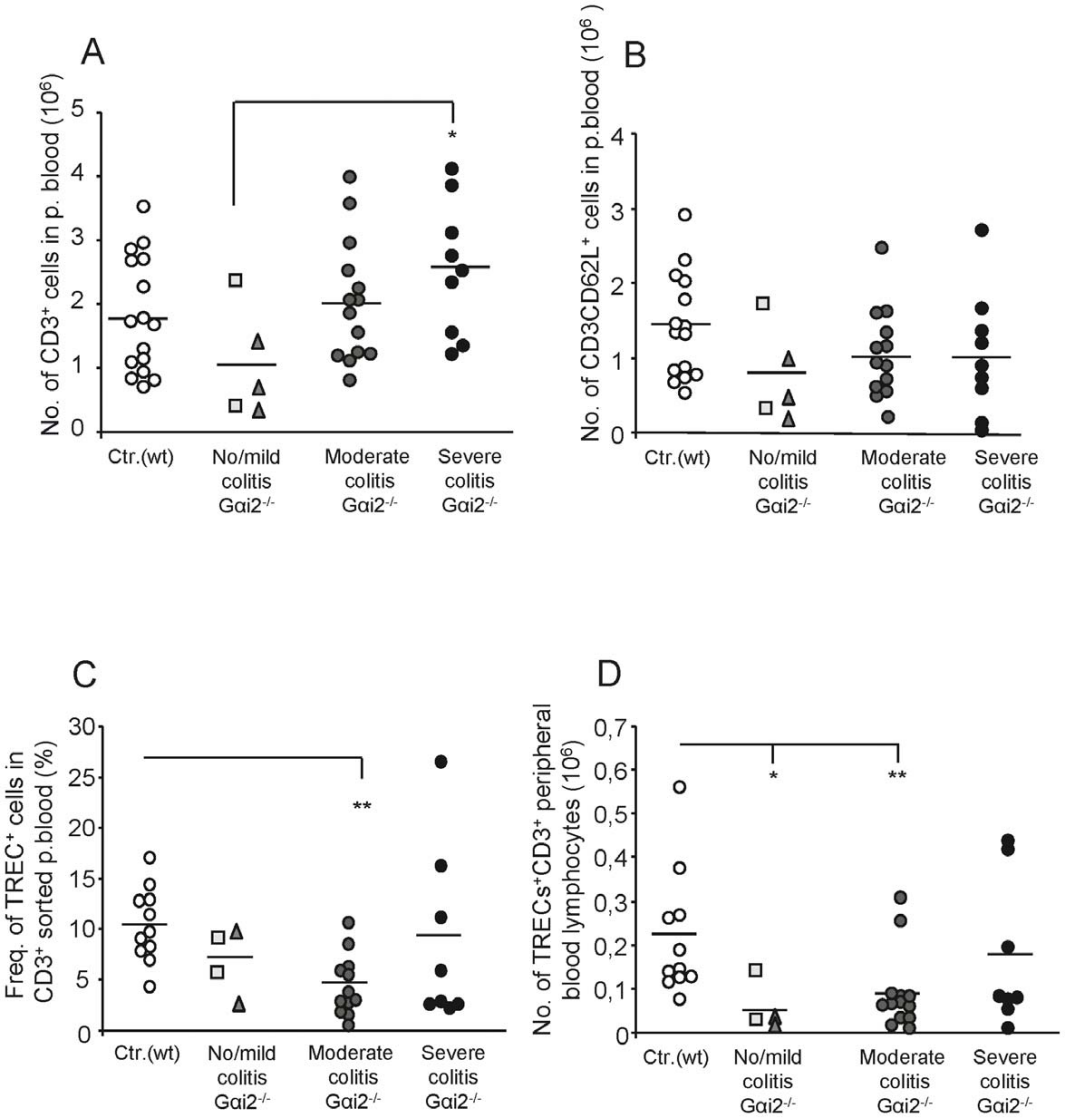
Decreased frequencies and numbers of TRECs in peripheral blood CD3^+^ T lymphocytes in colitic Gαi2^−/−^ mice. Peripheral blood was collected and the volume recorded from individual mice, whereafter Gαi2^+/−^ mice were pooled (3 mice/pool) and analysed, while peripheral blood from Gαi2^−/−^ mice was analysed individually. The control group of wt mice were 4–9 weeks old (n = 11–16, white circles) and the Gαi2^−/−^ mice were 4–8 weeks old (n = 4–14, gray/black symbols), grouped based on their colitis scores (no colitis = rectangles, mild colitis = triangles). PBMCs were isolated from peripheral blood and the frequencies of CD3^+^ T lymphocytes was analysed by flow cytometry. The number of CD3^+^ (A) and naïve T lymphocytes (B) are presented as total T lymphocytes of the entire blood volume in each mouse, 6–8 ml blood per 100 g body weight [40]. (C) The frequencies of TREC^+^ lymphocytes were analysed by real time-PCR on isolated DNA from flow cytometry sorted peripheral blood CD3^+^ T lymphocytes. (D) Numbers of TREC^+^ CD3^+^ peripheral blood lymphocytes, as calculated from data (A) and (C). Horizontal lines indicate the mean within the group. Statistical analysis was performed using the Mann-Whitney non-parametric test and values of p≤0.05 were considered significant. *p≤0.05; **p≤0.01 and ***p≤0.001 between Gαi2^−/−^ and wt mice.

Next, we evaluated if the observed dynamical changes of the Gαi2^−/−^ thymocytes maturation affect the number of T lymphocytes in peripheral blood. The number of naïve T lymphocytes and total amount of T lymphocytes in peripheral blood were calculated based on the fraction of CD3^+^CD62L^+^ cells or CD3^+^ cells (data not shown) of the total PBMCs population. Since the number of T lymphocytes as well as the number of TREC^+^ T lymphocytes in peripheral blood were stable with increasing age in 4–9 week old wt mice (no. of T lymphocytes and TREC^+^ T lymphocytes at age 4–5 w: 1.4±0.8×10^6^ and 1.6±0.9×10^5^; age 6–7 w: 2.3±0.6×10^6^ and 2.3±1.3×10^5^; and age 8–9 w: 1.9±1.1×10^6^ and 2.4±1.8×10^5^, respectively), the different age groups where grouped into a single wt control group. The control mice had a mean value of ∼1.8×10^6^ T lymphocytes and ∼1.5×10^6^ naïve T lymphocytes in peripheral blood (Fig. 3A and B). The amount of naïve T lymphocytes and total amount of T lymphocytes in Gαi2^−/−^ mice were comparable to the control mice, with one exception: The numbers of T lymphocytes were significantly lower in Gαi2^−/−^ mice with no/mild colitis compared to those with severe colitis (Fig. 3A). The mean fraction of TREC^+^ cells among the total T lymphocytes in peripheral blood of wt mice were found to be 10% (Fig. 3C), i.e. about half the fraction of TREC^+^ cells found in the wt mature thymocyte population (Fig. 2). This indicates that the peripheral blood T lymphocytes in wt mice have undergone on average one division since their most mature stage in thymus. Analysis of the Gαi2^−/−^ mice showed that only mice with moderate colitis had a significantly lower fraction of TREC^+^ peripheral T lymphocytes compared to control littermates (Fig. 3C).

We next calculated the total number of TREC^+^ T lymphocytes in peripheral blood by multiplying the total number of peripheral T lymphocytes with the fraction of TREC^+^ lymphocytes in the corresponding mice (Fig. 3D). This is an important parameter for estimating the daily egression of T lymphocytes since the number of TREC^+^ T lymphocytes in peripheral blood is independent on the T lymphocyte proliferation. The Gαi2^−/−^ mice with no/mild and moderate colitis had significantly lower numbers of TREC^+^ peripheral blood T lymphocytes compared to their control littermates (Fig. 3D).

### Reduced egression of thymocytes in Gαi2^−/−^ mice with no/mild and moderate colitis

**Figure 4:**
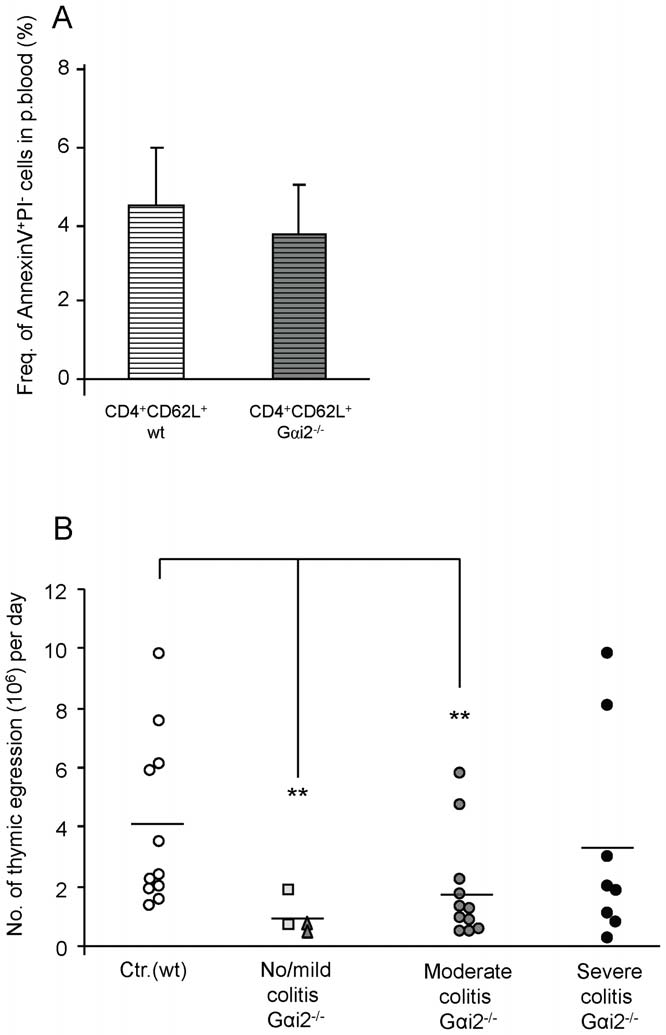
Reduced thymic egression in Gαi2^−/−^ mice with no/mild and moderate colitis. A. PBMCs were stained with CD4 and CD62L specific antibodies, as well as Annexin V and Propidium Iodide (PI) to analyse the frequency of naïve peripheral blood CD4^+^ T lymphocytes undergoing apoptosis. The bars depicts the mean frequency ± S.D. of apoptotic CD4^+^ T lymphocytes in wt mice (n = 6, light bar) and Gαi2^−/−^ mice (n = 5, dark bar (A). The daily egression was calculated by the following equation; 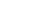, see Materials and Methods. (B) Four-nine weeks old wt mice (n = 11) and Gαi2^−/−^ mice (n = 4–11) indicate the individual numbers of thymocytes egressing per day. Gαi2^−/−^ mice were divided based on their stage of colitis (no colitis = rectangles, mild colitis = triangles). Horizontal lines indicate mean values within each group. Statistical analysis was performed using the Mann-Whitney non-parametric test and values of p≤0.05 were considered significant. *p≤0.05; **p≤0.01 and ***p≤0.001 between Gαi2^−/−^ and wt mice.

So far we had found only small differences in the total numbers of mature CD4 and CD8 SP thymocytes between Gαi2^−/−^ mice and control littermates (Fig. 1) as well as the fraction of TREC^+^ mature thymocytes (Fig. 2). However, a significant reduction in the number of TREC^+^ T lymphocytes in peripheral blood was demonstrated in Gαi2^−/−^ mice with no/mild and moderate colitis (Fig. 3D). To examine whether this resulted in a change in the daily egression of thymocytes to peripheral blood during progression of colitis, we next analysed the thymic egression rate of mature thymocytes. We first examined the fraction of apoptotic peripheral T lymphocytes in Gαi2^−/−^ mice and control littermates. Naïve peripheral blood CD4^+^ T lymphocytes from Gαi2^−/−^ and wt mice were stained with Annexin V and PI and analysed by flow cytometry. No significant difference in the fraction of cells staining positive for these apoptotic markers were found between mice with different macroscopic scoring, and therefore these mice were collected into a single group. The results showed comparable frequencies of apoptotic naive CD4^+^ T lymphocytes in wt and Gαi2^−/−^ mice (Fig. 4A). Next, we estimated the daily egression of thymocytes to peripheral blood. The calculations were based on the fraction of mature TREC^+^ thymocytes (Fig. 2), a death rate of 0.05/day of peripheral T lymphocytes as previously observed [28], [30], and the number of TREC^+^ T lymphocytes in the periphery (Fig. 3D) multiplied with 50 to account for that 1/50 of all the lymphocytes reside in the blood [28]–[29]. Since peripheral T lymphocytes of Gαi2^−/−^ and wt mice showed comparable fractions of cells that were positive for apoptotic markers, we assume equal death rates of Gαi2^−/−^ and wt mice. The calculations show that control mice at ages 4–9 weeks had an average thymic egression of 3.4–4.0×10^6^ thymocytes per day (age 4–5 w: 3.4±2.1×10^6^, age 6–7 w: 3.9±3.1×10^6^ and age 8–9 w: 4.1±3.6×10^6^), with a mean value of 4.0×10^6^ thymocytes/day (Fig. 4B). The Gαi2^−/−^ mice with no/mild and moderate colitis had a significantly reduced thymic egression rates, 0.87×10^6^ and 1.8×10^6^, respectively, compared to wt mice. However, mice with severe colitis showed thymic egression rates comparable to the control littermates.

## Discussion

Acute thymic atrophy has been reported in various situations; during infectious disease [31], pregnancy [5] autoimmune disease [6] and stress [32]. This identifies the thymus as a common target of several pathological and physiological situations. We have previously reported thymic atrophy in Gαi2 deficient mice and the resulting reduced frequencies and numbers of cortical thymocytes as well as reduced responsiveness to chemokines involved in thymocyte migration [11]. In this study, we show that the decreased numbers of DP thymocytes correlate well with the progression of colitis in the Gαi2-deficient mice. Zhang et al. have previously observed a dramatic reduction of the DP thymocytes with increasing age in Gαi2-deficient mice on a C57BL/6 background, which do not develop severe colitis [12]. Hence, this suggests that both the severity of colitis and the age of the Gαi2-deficient mice are likely to affect the number of DP thymocytes.

Interestingly, although the fraction of SP thymocytes increased during the progression of colitis their actual numbers remained largely unchanged. Thus, the increased fraction of SP thymocytes is a result of the reduced numbers of DP thymocytes and does not generally depend on changes in the actual numbers of SP thymocytes. Since the numbers of DP thymocytes, the main source for the SP population, were reduced, one would expect a reduction of the SP population as well. A number of factors may be involved to cause this aberrant thymocyte dynamics in the Gαi2-deficient mice. The DP thymocytes might die more frequently and to restore the SP thymocyte population the Gαi2^−/−^ SP thymocytes would need to proliferate more extensively and thus result in more diluted sjTRECs. However, we were unable to detect any differences in the frequencies of TREC^+^ mature thymocytes between the Gαi2^−/−^ mice and the control littermates. This is supported by previous cell cycle analyses of SP thymocytes demonstrating comparable fractions of cells being in the S+G2-M phase between Gαi2^−/−^ and wt SP thymocytes [12]. Hence, an increased proliferation of Gαi2^−/−^ SP thymocytes is not a compensatory mechanism to restore the number of SP thymocytes in the setting of reduced numbers of DP thymocytes. Another possibility is that Gαi2^−/−^ SP thymocytes do not die as frequently as the wt SP thymocytes. However, mature SP thymocytes have been shown to be largely resistant to cell death [33]. In addition, Jin and Wu reported that the lack of the Gαi2 protein resulted in reduced homing of blood-borne progenitor cells and caused arrest of thymocyte differentiation at the DN1 stage [34]. The observation that the death rate and the proliferation rate of Gαi2^−/−^ DP thymocytes were comparable to wt mice [8], [12] suggests that the most likely mechanisms generating decreased numbers of DP but comparable numbers of SP thymocytes in Gαi2^−/−^ compared to control littermates are: (i) an accelerated transition rate from DP to SP thymocytes, (ii) a reduced recruitment rate from DN to DP and (iii) that the SP thymocytes reside longer in the thymus, i.e. egress more slowly, thus enabling accumulation of SP thymocytes. In support of this, Zhang et al. has previously proposed that Gαi2^−/−^ mice have an accelerated transition from DP to SP thymocytes [12], and our data indeed support this theory. In addition, in our previous work we demonstrated an accumulation of the most mature thymocytes in pre-colitic Gαi2^−/−^ mice before egress from the thymus, supporting the latter explanation [11]. The lack of the Gαi2 protein may also affect the chemotactic response within the thymus. We have previously reported that the numbers of cortical DP as well as early medullary SP thymocytes migrating towards the medulla in response to CXCL12 were decreased [11]. In addition, this decreased migratory response could not be explained by decreased expression of CXCR4 [11]. The spontaneous mobility, i.e. without chemokines was, however, increased in pre-colitic mice, supporting the hypothesis of an accelerated transition rate from DP to SP thymocytes [11]. In addition, our calculations of the thymic egression rate also showed that Gαi2^−/−^ mice with no/mild and moderate colitis have a reduced egression rate. The predicted slower egression rate during no/mild and moderate colitis is due to the low number of TREC^+^ T lymphocytes observed in peripheral blood since neither of the other parameters showed a significant difference. Even though the method we use to calculate the export rate is a rough estimation, we obtained similar egression rates as the ones previously reported in mice, [30], [33], [35]–[37] ranging from 1–5 million per day. Our calculations of the daily thymocyte egression rate in 4–9 week old wt mice were ∼4 million or ∼3% of the total numbers of thymocytes. A reduced export rate during aging was not observed in the wt mice in this study, which is consistent with the report by Sempowski et al., who only observed a declined TREC^+^ fraction of mouse peripheral T lymphocytes after the age of 35 weeks [22]. In addition, the frequency of sjTREC containing mature thymocytes has previously been reported to be around 10–15% in healthy individuals [38]–[39], which is similar to what we obtained. The mature CD4 or CD8 SP thymocytes in the wt mice had an equal percentage of TREC^+^ cells. This suggests that CD4 and CD8 SP thymocytes undergo the same number of divisions before they enter the periphery, which is supported by kinetic studies of thymocyte development [33]. The method we used to calculate the export rate is one of the most commonly used methods used to predict the export rate [16] and the values for the individual parameters also seem to be in line with what others have reported. The only parameter that we did not measure directly was the death rate of TREC^+^ T lymphocytes. Since the mice are kept in a pathogen free environment and that a considerable fraction of T lymphocytes are not specific for antigens involved in colitis, most of the TREC^+^ T lymphocytes will remain in their naïve state. In addition, the frequency of peripheral naïve T lymphocytes undergoing apoptosis were comparable between Gαi2^−/−^ and wt control mice, further supporting our assumption of equal death rates of TREC^+^ T lymphocytes in Gαi2^−/−^ and wt mice.

The finding that mice with severe colitis have thymic egression rates comparable to their control littermates was unexpected. However, these mice have reached a critical disease stage where the homeostatic balance might be completely lost. Since these mice will have leakage of plasma fluids into peripheral tissues, the appreciated blood volume of them might be over estimated. A reduced blood volume in our calculations will reduce the amount of TREC^+^ T lymphocytes and consequently the egression rate. In addition, there is an uncertainty in the data since two distinct mice show about five-fold higher egression rate compared to the other mice in this group. Hence, further investigations are needed to confirm the thymic egression rates in mice with severe colitis.

In conclusion, we propose a possible chain of events that occurs during the progress of colitis in the Gαi2^−/−^ mice. The reduced number of DP thymocytes is due to an accelerated transition from DP to SP thymocytes and/or a reduced transition from DN to DP thymocytes. In order to keep the SP cells at a normal level even though the number of DP thymocytes is reduced, the SP thymocytes reside longer within the thymus before they egress. Thus, this results in a lower daily production by the thymus. Future studies revealing the exact mechanisms behind the altered transition rates in colitic mice will provide important information about the thymic atrophy during colitis.
